# Multistate occupancy modeling improves understanding of amphibian breeding dynamics in the Greater Yellowstone Area

**DOI:** 10.1002/eap.1825

**Published:** 2019-01-02

**Authors:** William R. Gould, Andrew M. Ray, Larissa L. Bailey, David Thoma, Rob Daley, Kristin Legg

**Affiliations:** ^1^ Applied Statistics Program New Mexico State University Box 30001/MSC 3CQ Las Cruces New Mexico 88003 USA; ^2^ National Park Service Greater Yellowstone Network 2327 University Way, Suite 2 Bozeman Montana 59715 USA; ^3^ Department of Fish, Wildlife and Conservation Biology and the Graduate Degree Program in Ecology Colorado State University 1474 Campus Delivery, Fort Collins Colorado 80523 USA; ^4^ National Park Service Northern Colorado Plateau Network 2327 University Way, Suite 2 Bozeman Montana 59715 USA

**Keywords:** breeding, climate drivers, frogs, modeling, monitoring, multistate, national parks, occupancy, wetland dynamics

## Abstract

Discerning the determinants of species occurrence across landscapes is fundamental to their conservation and management. In spatially and climatologically complex landscapes, explaining the dynamics of occurrence can lead to improved understanding of short‐ vs. long‐term trends and offer novel insight on local vs. regional change. We examined the changes in occupancy for two species of anurans with different life histories over a decade using hundreds of wetland sites in Yellowstone and Grand Teton National Parks. To account for the joint dynamics of wetland drying and amphibian breeding, we adopted a multistate occupancy model as a means to investigate mechanistic relationships of observed occurrence patterns with climatological drivers of wetland hydrologic variability. This approach allowed us to decompose occupancy dynamics into habitat changes caused by wetland drying and amphibian breeding activity, conditional on available water and previous breeding state. Over our 10‐yr time series, we observed considerable variability in climate drivers and the proportion of dry wetlands. Boreal chorus frogs (*Pseudacris maculata*) were more responsive to changes in wetland inundation status than Columbia spotted frogs (*Rana luteiventris*), as indicated by higher breeding colonization probabilities under favorable (wet) conditions. Both species had high probabilities of breeding persistence in permanently inundated wetlands with prior breeding. Despite the absence of multi‐year drought in our time series, mechanistic relationships described here offer insights on how future climate variation may result in reduced and/or shifted occurrence patterns for pond‐breeding anurans in the Greater Yellowstone Area. Further, our modeling approach may prove valuable in evaluating determinants of occurrence for other species that are dependent on wetlands or other dynamic habitats.

## Introduction

The ability to explain the pattern of species occurrence is important for the conservation and management of biological resources (Rondinini et al. 2006, Abell et al. 2008). Among the determinants of species distributions are the presence of suitable habitat (landscape‐scale attributes; Kolozsvary and Swihart 1999), habitat features at regional or local scales (Austin et al. 1990, Price et al. 2005), species interactions (e.g., predation; Kuang and Chesson 2008), disease (Muths et al. 2008), land use (Hilty et al. 2006, Fox et al. 2014), and climatic conditions (Luoto et al. 2006). All of these determinants are spatially and temporally dynamic further influencing multi‐year patterns of species occurrence. A long‐standing paradigm in ecology is that species occurrence is limited by climate at broader geographic scales, habitat availability at regional scales, and land use activities may impact finer scales (Pearson et al. 2004, Wiens 2009).

In response to demonstrated global (Houlahan et al. 2000, Stuart et al. 2004) and continental‐scale declines (Adams et al. 2013), researchers have investigated the influence of several determinants (e.g., disease, drought, and human influences) on global amphibian diversity (Hof et al. 2011) and trends in amphibian occurrence in North America (Grant et al. 2016, Davis et al. 2017, Miller et al. 2018). To date, no single stressor has been universally linked to amphibian decline. Grant et al. (2016) concluded that regional and/or local analyses will be important to discern the drivers behind the observed declines and, ultimately, inform conservation strategies. With this in mind, we examined 10 yr of amphibian occurrence data in two national parks to identify factors most related to the dynamic patterns of amphibian breeding occurrence.

There is mounting evidence that climate‐related weather characteristics (hereafter referred to as climate drivers) should be explicitly considered when examining amphibian occupancy dynamics (Cayuela et al. 2012, Ray et al. 2016, Greenberg et al. 2017, Miller et al. 2018). Wetlands are often the principal breeding habitat for amphibians and these habitats are dynamic, fluctuating between wet and dry states depending on annual and seasonal meteorological conditions. To integrate meteorological conditions and wetland status and their effect on amphibian breeding occupancy, we adopted a multistate occupancy approach (MacKenzie et al. 2009) that has proven useful in other amphibian systems (Walls et al. 2013, Davis et al. 2017). Specifically, we used current and lagged estimates of precipitation, evapotranspiration, and runoff to examine how these climate drivers influence a decade of annual wetland patterns across our study area. This modeling approach improves our ability to identify functional relationships between spatially and temporally available aquatic habitat and amphibian breeding therein to inform future predictions under various climate scenarios.

Recent studies highlight species‐specific differences in the influence of broad scale (e.g., climate change) and local scale factors on amphibian demographic rates (Mazerolle et al. 2005, Muths et al. 2017). In a comparison of 11 species from 31 long‐term data sets, Muths et al. (2017) concluded that demographic rates varied due to a combination of climate drivers and interspecific differences in species life history characteristics. Cayuela et al. (2017) have also suggested that life history theory can contribute to our understanding of interspecific differences to large‐scale processes such as the North Atlantic Oscillation. These authors predicted recruitment would be more robust to environmental variation for species with short life spans (referred to as fast species) and that survival would be buffered for longer‐lived species (referred to as slow species.)

Occupancy‐related vital rates, including probabilities of local colonization and local persistence (or conversely, local extinction), are different than vital rates for age‐structured populations (e.g., recruitment and survival probabilities). However, climate, local factors, and life history characteristics are likely to influence amphibian occupancy and breeding dynamics in ways that are consistent with demographic‐based life history theory (Davis et al. 2017). For example, if wetland habitat remains suitable among years, we would expect the breeding probability for all species to be high, unless predators such as fish inhabit these permanent wetlands. In contrast, shorter‐lived species are more likely to disperse and are expected to have a higher probability of colonizing (breeding in) newly available breeding habitats (i.e., habitats that are not suitable each year). Cayuela et al. (2016b) refer to several studies that describe a “colonizer syndrome” in populations that links high dispersal rates with shorter‐lived species in interspecific and intraspecific studies. We focus on two species that conform to these differing life histories (short lived vs. long lived) and explore how their breeding dynamics vary across a collection of dynamic wetland habitats.

## Methods

### Study area

Grand Teton National Park, John D. Rockefeller, Jr. National Memorial Parkway, and Yellowstone National Park (combined area ~10,300 km^2^) are adjacent national park units located primarily in northwest Wyoming. Hereafter, Grand Teton National Park refers to lands within Grand Teton National Park and the adjacent John D. Rockefeller, Jr. National Memorial Parkway. Elevation ranges from 1,600 m in the lowest portion of Yellowstone to >4,000 m in the Teton Range. Vegetation of the two parks includes sagebrush and grasslands at lower elevations, conifer forests at middle and upper elevations, and deciduous trees, willows, grass–sedge, and forbs in moist areas (Ray et al. 2016). The climate is characterized by long, cold winters and brief, cool summers, with considerable spatial heterogeneity in annual precipitation (25 cm to >200 cm; Wright and Gallant 2007). Snow provides the main source of surface water (Pederson et al. 2011). Wetlands of the study area are diverse in size, depth, and hydroperiod, and are most prominently influenced by local soil permeability (Elliot and Hektner 2000). Isolated, palustrine wetlands are the predominant wetland type and constitute ~3% of the area of the two parks (Gould et al. 2012). Elevation of surveyed wetlands ranged from 1,947 to 2,789 m.

### Amphibian community

Western tiger salamanders (*Ambystoma mavoritum*), western toads (*Anaxyrus boreas*), and plains spadefoot (*Spea bombifrons*) occur in the study area, but have very restricted ranges (e.g., plains spadefoot) or are relatively uncommon (tiger salamander and toad have a long‐term occurrence <10% at monitored wetlands). We focused on two frog species that are widespread in both parks: boreal chorus frog (*Pseudacris maculata*; PSMA), and Columbia spotted frog (*Rana luteiventris*; RALU). Boreal chorus frogs typically live 3–7 yr reproducing in their first or second year (Tordoff and Pettus 1977, Muths et al. 2016). Boreal chorus frogs across the region use a variety of ephemeral and shallow water habitats that contain abundant emergent vegetation for breeding (Bartelt et al. 2011) and typically move away into terrestrial environments immediately after the breeding season (Koch and Peterson 1995). Columbia spotted frogs are highly aquatic and typically use deeper, permanent water bodies for breeding (Gould et al. 2012, Ray et al. 2016). After breeding, spotted frogs often remain at breeding sites, but sometimes move to neighboring aquatic habitats to forage and overwinter (Bull and Hayes 2001, Pilliod et al. 2002). Spotted frogs live 7–13 yr (Turner 1960, Engle 2001) and, in Yellowstone National Park, reach sexual maturity in 4–6 yr (Turner 1960); females of this species likely breed every 2 or 3 yr (Turner 1958). Although variable across their range, chorus frogs and spotted frogs typically metamorphose in mid to late summer (Koch and Peterson 1995). Neither species has larvae that overwinter at breeding sites.

### Survey methods and climate covariates

We randomly sampled a total of 34 catchments (portions of watersheds averaging ~200 ha in size) previously characterized as high to medium quality based on the amount and permanency of wetlands as described in Ray et al. (2016). Most catchments were surveyed each year (*n *= 28–32). Two catchments were discontinued the last two years as a measure of protection for species of conservation concern (e.g., grizzly bears and trumpeter swans) at sensitive times. Following these discontinuations, two catchments were added in 2012 and one more was added beginning in 2014 to maintain a comparable among‐year sample size. The number of wetlands visited each year (*n *= 246–326) varied due to environmental conditions and in some cases, new unmapped wetlands were found that were not known to exist previously. All wetlands included in the analysis were surveyed (and therefore contained water) during at least two years between 2006 and 2015. Wetlands with only a single visit were excluded, as were sites that were associated with large lakes (e.g., Jackson or Yellowstone lakes), seeps or other sloping wetlands that lack habitat necessary for pond‐breeding amphibians, and sites that were inaccessible due to geothermal hazards. Wetland survey methods are described in more detail in Ray et al. (2016). In brief, two observers surveyed each wetland independently on a single visit using visual observation and dip‐netting to detect amphibian breeding evidence (egg masses, larvae, etc.). We separated replicate surveys by ~15 min allowing no communication between observers until after survey completion. These methods are similar to that described in Zipkin et al. (2012) and Groff et al. (2017). On each visit, we recorded site‐specific covariates (habitat characteristics) such as wetland size (m^2^), percent cover of emergent vegetation, and maximum depth (<0.5 m, 0.5–1 m, 1–2 m, >2 m).

**Figure 1:**
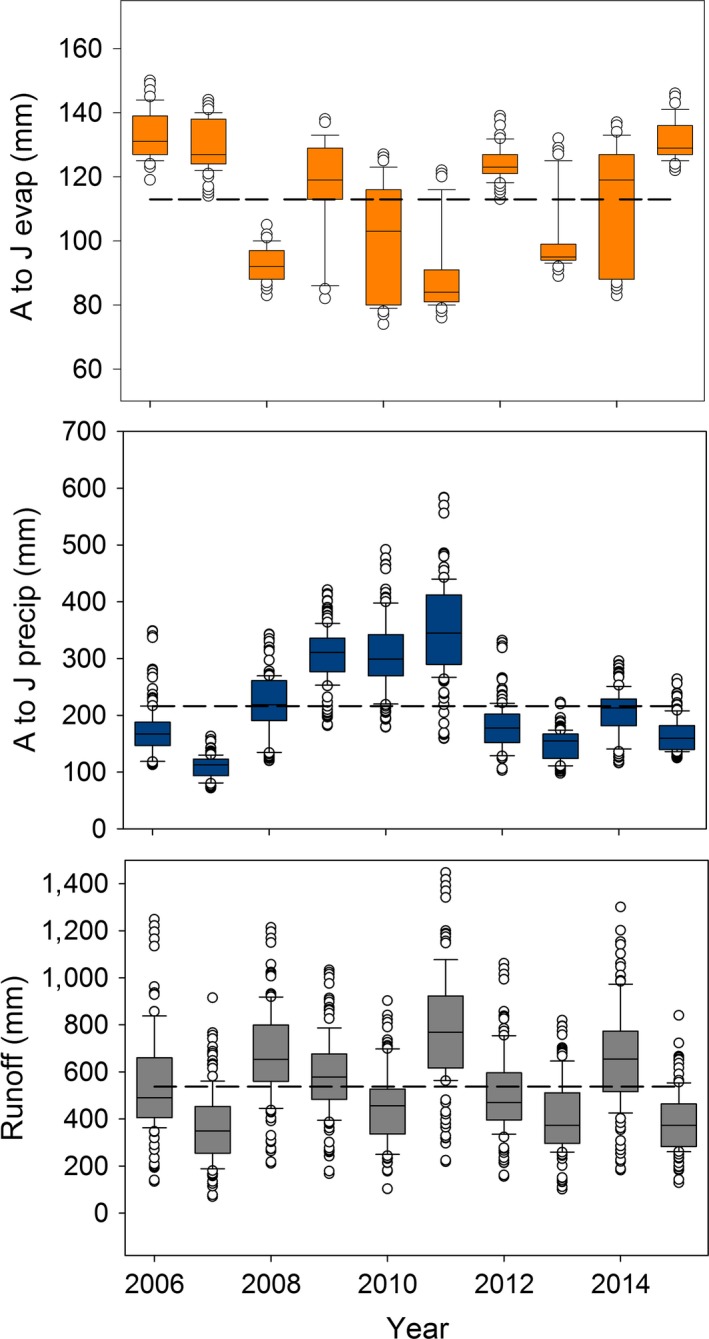
Annual variation in climate drivers for all monitored wetlands. Precipitation (precip; mm) and evapotranspiration (evap; mm) were summed over three months between April and June (A to J). Runoff was estimated on an annual basis. The dashed line represents the 10‐year average across all sites. The box plots demonstrate the interquartile range with the mid line indicating the median. Whiskers represent the 10th and 90th percentiles of the distribution.

To estimate annual site‐specific climatological covariates, we used high resolution (1 km^2^) spatially interpolated Daymet gridded climate data sets (Thornton et al. 2017) to summarize daily estimates of air temperature and precipitation (precip) for all monitored wetlands within the study area. Precipitation (mm) was summed over three months between April and June because this period was most likely to affect wetland persistence during amphibian breeding, oviposition, and larval development (Murphy et al. 2010). We combined gridded estimates of daily meteorological parameters with a water balance model (Lutz et al. 2010) to estimate evapotranspiration (evap; mm) and runoff (RO; mm) at the monthly time step (Ray et al. 2016). Evapotranspiration rates were also estimated from April to June. Snow melt runoff was estimated on an annual basis because it serves as the primary source of moisture filling high elevation wetlands (Corn 2005). The ranges of these covariates are illustrated in Fig. 1 to show the spatial and temporal variation at our wetlands. We defined wet years as those with notably lower evapotranspiration and higher runoff (2008 and 2011). Years with notably higher evapotranspiration and lower runoff are referred to as dry years (2007 and 2015). We considered current‐year values and one‐year lagged values of these covariates (separately) when modeling wetland dynamics because antecedent weather conditions may contribute to and/or be useful in describing current wetland conditions (Cayuela et al. 2014, Lee et al. 2015). We assessed covariates for multicollinearity by considering Pearson correlation coefficients (*r*) and eliminated covariates that had high correlations (*r *≥ 0.60) from appearing in the same model.

### Parameter estimation and model selection

Our multistate approach allowed wetlands to be in one of three mutually exclusive states in a given year. Typical applications of multistate occupancy models have one unoccupied state and two occupied states (Băncilă et al. 2017). In our system, we define two unoccupied states, one when a wetland is dry (*m *= 0) and another when the wetland is wet but does not support breeding by the target amphibian (*m *= 1). Our single occupied state (*m *= 2) is conditional on water presence and represents breeding by the target amphibian (breeding occupancy). Using the conditional parameterization of the multistate model, the initial state probability vector is defined as ϕ_0_ **= [(1 – ψ) ψ(1 − *R*) ψ*R*], where ψ is the probability of a wetland being wet in the first year of the study and *R* is the probability of being in state 2 (occupied with breeding), given that a site is wet. Annual transitions between our three states are governed by two types of dynamic parameters (MacKenzie et al. 2009): ψ^*m*^[*t*], is the probability that a wetland in state *m* in year *t* is wet in year *t* + 1, and R^*m*^[*t*] is the conditional probability that a wetland in state *m* in year *t* supports breeding in year *t *+ 1, given the wetland is wet in year *t *+ 1. The term “wetland inundation” is defined hereafter as the process in which dry sites (state *m *= 0) transition to holding water (state *m *= 1 or 2). The probability of a wetland being dry, given its previous state *m*, is calculated as 1 − ψ^*m*^(*t*).

Detection probabilities are conditional on the true state of the site and are given as


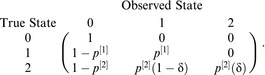


In our application, *p* ^[*m*]^ is the probability of detecting water, given a site is wet (states *m *= 1 or 2) and δ is the probability of detecting breeding given the site is wet and has breeding. In our case, detection probability of water at a wet site is 1 (i.e., *p* ^[1]^ **= *p* ^[2]^ **= 1), so the detection matrix reduces to


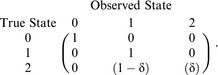


Breeding evidence at wet sites included the presence of eggs, larvae, or recently metamorphosed juveniles. This metric was chosen for occurrence because breeding activity suggests maintenance of a breeding population as opposed to adults moving across an area. Chorus frogs and spotted frogs were analyzed separately and, for breeding dynamics, we used the top models from Ray et al. (2016) as a baseline from which we constructed our model set.

We evaluated 31–39 models (Appendix  S1: Tables S1 and S2) for wetland and breeding occupancy dynamics using information theoretic methods as described in Burnham and Anderson (1998) and a likelihood approach in Program MARK (White and Burnham 1999). We used a multistep model building process in constructing the model set to avoid a model set with all possible combinations of factors. First, we assumed state and time varying models for wetland transition probabilities, ψ^*m*^(*t*), and conditional breeding occupancy probabilities, R^*m*^(*t*), while investigating models for detection. We assumed detection probability was constant between surveys within a season given their close proximity in time and previous work that demonstrated little support for survey‐specific differences (Gould et al. 2012). Detectability was modeled as constant among years, δ(·), year‐specific, δ(*t*), related to annual, site‐specific vegetation cover, δ(veg), or as an additive function of year and site‐specific vegetation cover averaged across years, δ(Vegave *+ t*), using different annual intercepts but assuming the same relationship (slope) with vegetation cover among years.

The top model for detection probability according to Akaike's Information Criterion corrected for small sample size (AIC_c_; Hurvich and Tsai 1989) was retained when subsequently modeling wetland dynamics. Specifically, we considered models where state‐specific wetland dynamics were constant over years (ψ^*m*^(*·*)), related to climate covariates that vary spatially and temporally (ψ^*m*^(covariate), and related to climate drivers that vary spatially, by averaging values over years, with additive year‐specific effects, ψ^*m*^(Covariateave + *t*). Wetland dynamics were modeled as an interaction between previous hydrological‐occupancy state (*m*) and unnecessary climate covariates, with one exception. We also considered models where wetland transition probabilities were related to prior hydrological state only (dry or wet), independent of amphibian occurrence, by constraining/equating transition probabilities for states 1 and 2, ψ^1=2^(*t*) and ψ^1=2^(·).

Using the top model for wetland dynamics, we investigated different models for conditional breeding probability. Specifically, we modeled state‐specific breeding probabilities with yearly variation, *R* ^*m*^(*t*), spatially and time varying covariates, *R* ^*m*^(covariate), and additive effects of year and one or two climate drivers averaged over years, e.g., *R* ^*m*^(Covariateave + *t*). Conditional breeding probability was modeled as an interaction between previous hydrological‐occupancy state (*m*) and year and/or climate covariates. We suspected that breeding probability might be higher for those wetlands that supported breeding/reproduction the previous year (*m *= 2), relative to other wetlands, so we considered a model structure where *R* ^0=1,2^(*t*). We also considered models where conditional breeding was influenced by only the previous hydrological state (dry or wet), *R* ^0,1=2^(*t*), but lacking any support, did not pursue all other model possible combinations with this constraint. When the top ranked model had substantial support (Akaike weight >0.90), we made inference from the top model, otherwise model averaged estimates are provided.

### Assumptions and caveats

Our dynamic multistate occupancy model has several assumptions. First, we assume our surveys were timed appropriately so if breeding occurred, there was a nonzero chance of detecting eggs and/or larvae. Surveys are timed to take place after amphibian egg laying and prior to larval metamorphosis while considering such factors as elevation. This period normally begins in mid‐June and lasts about eight weeks, but the survey schedule is applied with flexibility to allow for late snowmelt, drought, or other unusual weather patterns that will occur in some years.

Our use of multiple states, climate drivers, and habitat covariates is meant to diminish the effects of heterogeneity on parameter estimates. We assume breeding detections and detection histories at each location are independent. The former condition is addressed by the sampling protocol (Bennetts et al. 2013) while the latter is a function of spatial correlations, breeding ecology, and distances individuals are likely to travel. In addition, we assume that the target species was never falsely detected. Our amphibian system has few species whose eggs masses and larvae are easy to differentiate, so the likelihood of false detections is low. For those wetlands that we were unable to visit in a given year, we assume that the transition probabilities for these sites are the same as for wetlands that were visited (MacKenzie et al. 2006). In general, if these assumptions are not met, then estimators may be biased, although the extent of the bias has received little attention (but see Miller et al. 2015).

## Results

### Detection probability

Our top model for detection probability was year specific and indicated a positive association with the average vegetation cover at each site for both species (Table 1). For chorus frogs, year‐specific detection probabilities at a wetland with average vegetative cover varied between 0.74 and 0.93 (SE range: 0.02–0.05), except for a notably lower value in 2010 ($\hat{p}=0.60$; SE* *= 0.03). For spotted frogs, the second ranked model was year‐specific and did not include a relationship with vegetation cover (Table 1). Model averaged detection probabilities for spotted frogs were similar to chorus frogs, ranging between 0.73 and 0.86 among years for wetlands with average vegetative cover, except for a notably low value in 2010 ($\hat{p}=0.49$; SE* *= 0.05).

**Table 1:** Model selection results for detection probability for (a) boreal chorus frogs and (b) spotted frogs at wetland sites within the Greater Yellowstone Ecosystem

Model	AIC_c_	ΔAIC_c_	AIC_c_ weight	*K*	Deviance
(a) Boreal chorus frogs					
δ (Vegave* *+ *t*)	6,829.88	0.00	1.00	67	6,692.67
δ (*t*)	6,846.96	17.08	0.00	66	6,711.84
δ (veg)	6,849.02	19.14	0.00	58	6,730.62
δ (·)	6,884.31	54.44	0.00	57	6,767.99
(b) Spotted frogs					
δ (Vegave* *+ *t*)	5,814.29	0.00	0.61	67	5,677.08
δ (*t*)	5,815.21	0.92	0.39	66	5,680.09
δ (veg)	5,831.45	17.16	0.00	58	5,713.05
δ (·)	5,835.67	21.38	0.00	57	5,719.35

*Notes*

Wetland and breeding transition probabilities were state (*m*) and year (*t*) specific, ψ^*m*^(*t*)R^*m*^(*t*), for all models. Covariates are abbreviated as follows: veg represents site‐specific vegetation cover that varies by year, Vegave represents the average vegetation cover at sites across years. Model selection information including Akaike's Information Criterion corrected for small sample size (AIC_c_), the difference in AIC_c_ from the top ranked model (ΔAIC_c_), model weights, number of parameters (*K*), and deviance are shown for each model.

### Wetland dynamics

**Figure 2:**
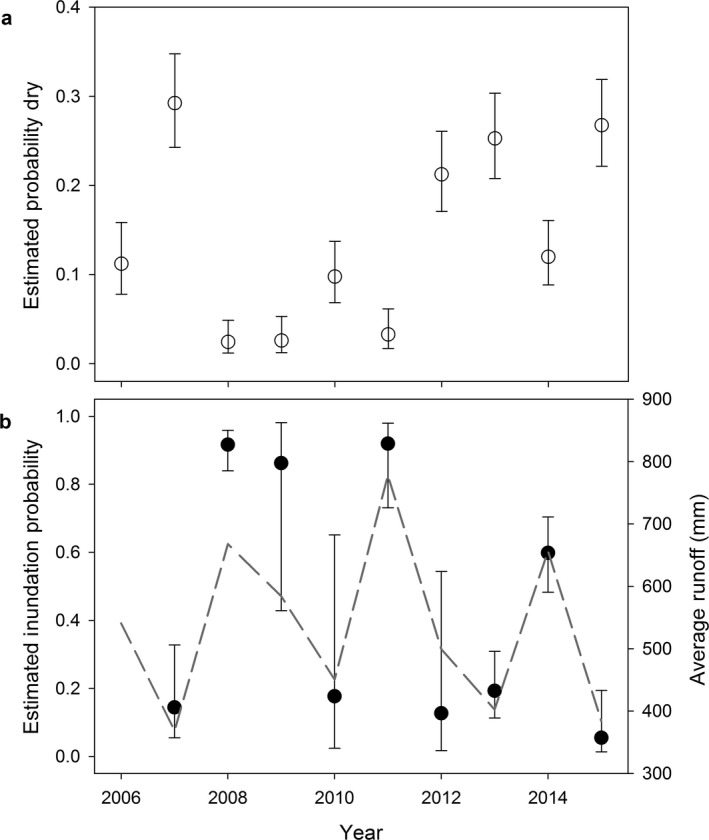
(a) Derived estimates of the proportion of dry wetlands (i.e., probability [*m *= 0]) over a 10‐yr period in Yellowstone and Grand Teton National Parks and (b) estimated annual inundation probabilities of dry wetlands in the prior year, ψ^0^(*t*). Estimates are given for the best supported model, using the average precipitation value across wetlands and years from Program MARK. The average runoff by year for surveyed sites (dashed line) is also given as a means for distinguishing “dry” vs. “wet” years. Error bars indicate approximate 95% confidence intervals.

Over our decade‐long time series, we made 2,910 site visits and recorded wetland status and evidence of amphibian breeding. Dry wetlands represented a minority of observations, but were documented each year. Wetland dynamics varied among years and wetlands (Table 2), but were not modeled well with spatial and temporal covariates alone (e.g., ψ^*m*^(covariate) structures; Appendix  S1: Tables S1, S2). Derived annual proportion of dry wetlands (i.e., probability that *m *= 0) varied among years, ranging from 2.6% in 2008 (wet year) to 29.5% in 2007 (dry year; Fig. 2a). Unoccupied wetlands account for the majority of these wetland dynamics, specifically sites that only have water in years with above average precipitation and runoff (Fig. 2b). The probability of wetland inundation (ψ^0^(*t*)) was <0.20 in most years, but was >0.90 in wet years (2008, 2011; Fig. 2b). The probability of retaining water at unoccupied wetlands (ψ^1^(*t*)) varied from 72% to 100%. Wetlands occupied by amphibians were more hydrologically stable and remained wet, even in dry years (ψ^2^(*t*) values ranged between 81% and 100%). In many ways, the presence of breeding amphibians serves as an indicator of local wetland stability in our study area. Interestingly and contrary to our expectation, average precipitation (over the decade) at wetlands was negatively associated with the probability of dry wetlands becoming inundated, ψ^0^(*t*), or wetlands remaining wet, ψ^1^(*t*) and ψ^2^(*t*). Although our use of time varying climate covariates were not as supported as the more generic year‐specific models (Appendix  S1: Tables S1, S2), the wetland inundation (ψ^0^) and water retention (ψ^1^ and ψ^2^) probabilities were positively associated with runoff and precipitation and negatively associated with evapotranspiration, as one would expect (e.g., see Fig. 2b).

**Table 2:** Model selection results (ΔAIC < 10) for state‐specific wetland transition probabilities ψ^*m*^ for potential (a) boreal chorus frogs and (b) spotted frogs breeding sites within the Greater Yellowstone Ecosystem

Model	AIC_c_	ΔAIC_c_	AIC_c_ weight	*K*	Deviance
(a) Boreal chorus frogs					
ψ^*m*^(Precipave* *+ *t*)	6,827.02	0.00	0.56	70	6,674.73
ψ^*m*^(Evapave* *+ *t*)	6,828.72	1.70	0.24	70	6,685.22
ψ^*m*^(*t*)	6,829.88	2.86	0.13	67	6,692.67
ψ^*m*^(ROave* *+ *t*)	6,831.16	4.14	0.07	70	6,687.66
(b) Spotted frogs					
ψ^*m*^(Precipave* *+ *t*)	5,813.73	0.00	0.45	70	5,670.22
ψ^*m*^(*t*)	5,814.29	0.56	0.37	67	5,677.08
ψ^*m*^(ROave* *+ *t*)	5,817.09	3.36	0.08	70	5,673.58
ψ^*m*^(Precipave* *+ Evapave* *+ *t*)	5,817.43	3.71	0.07	73	5,667.62
ψ^*m*^(Evapave* *+ *t*)	5,818.72	4.99	0.03	70	5,675.21

*Notes*

Models include year and state‐specific conditional breeding probabilities (R^*m*^(*t*)) and the best supported detection structure, δ(Vegave* *+ *t*). Prior wetland states (*m*) were modelled independently and *t* represents year‐specific parameters. Spatial variation among wetlands was measured as a function of covariates: Vegave represents the average vegetation cover at sites across years, and Evapave, Precipave, and ROave represent the average values of evapotranspiration, precipitation, and runoff. Model selection information including Akaike's Information Criterion corrected for small sample size (AIC_c_), the difference in AIC_c_ from the top ranked model (ΔAIC_c_), model weights, number of parameters (*K*), and deviance are shown for each model.

### Chorus frog dynamics

**Figure 3:**
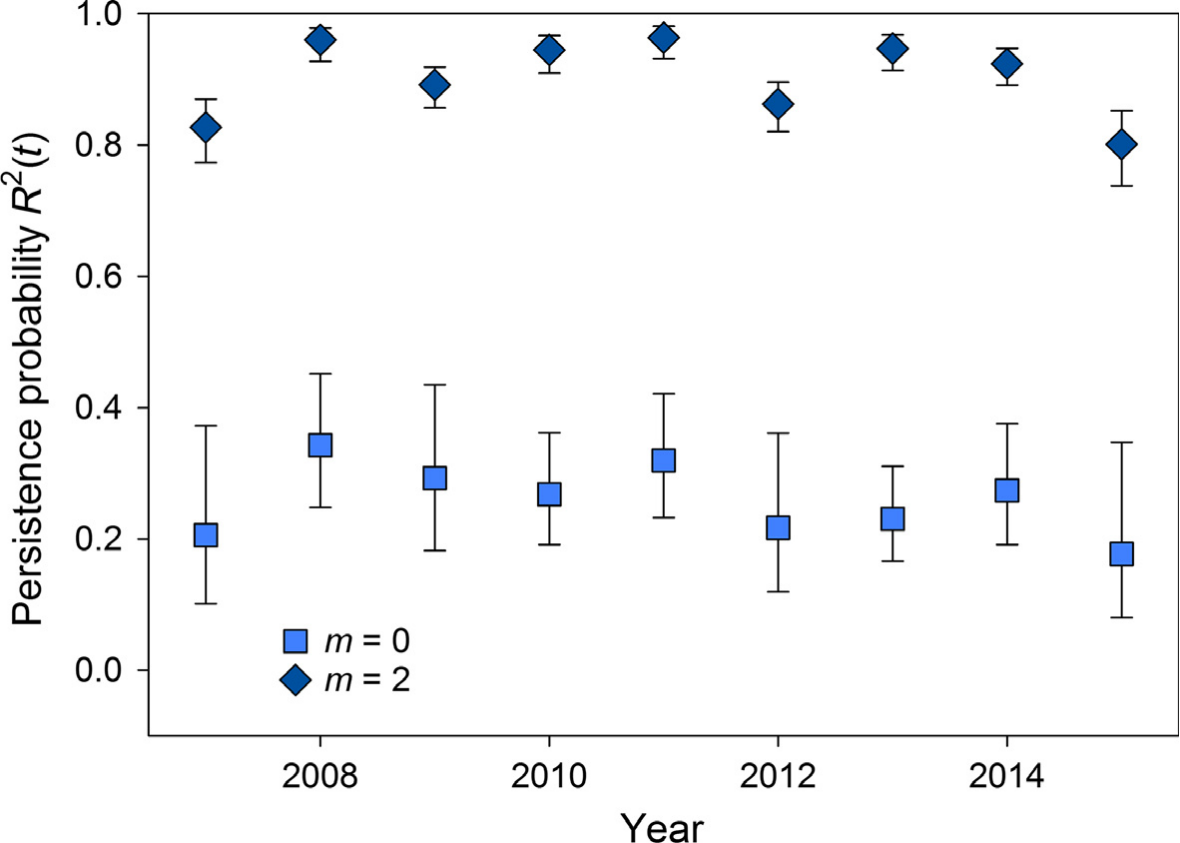
Estimated conditional breeding probabilities for chorus frogs, R^*m*^(*t*), at previously dry sites (state *m *= 0) and at previously occupied sites (state *m *= 2). Estimates are given for the best supported model, ψ^*m*^(Precipave* *+ *t*) R^*m*^(evap* *+ depth* *+ veg) δ(Vegave* *+ *t*), using the average covariate values in each year. Error bars indicate approximate 95% confidence intervals.

Based on the top model and year‐specific average covariate values (Table 3), chorus frog conditional breeding probabilities for previously dry sites (*R* ^0^(*t*)) were highest in wet years (2008, 2011) and lowest in dry years (2007, 2015; Fig. 3). Further, conditional breeding probabilities were higher for previously dry sites than previously wet sites without breeding, which ranged from 7% to 8%. Wetlands with breeding in the previous year had high probabilities of chorus frog breeding persistence in the following year (*R* ^2^(*t*) between 80% and 96%) with the lowest estimates occurring in dry years in 2007 and 2015 (Fig. 3).

**Table 3:** Conditional breeding transition models (ΔAIC < 10) for (a) boreal chorus frogs and (b) spotted frogs at wetland sites within the Greater Yellowstone Ecosystem

Model	AIC_c_	ΔAIC_c_	AIC_c_ weight	*K*	Deviance
(a) Boreal chorus frogs					
*R* ^*m*^(evap* *+ depth* *+ veg)	6,784.40	0.00	0.94	55	6,672.25
*R* ^*m*^(evap* *+ veg)	6,789.89	5.48	0.06	52	6,683.96
(b) Spotted frogs					
*R* ^*m*^(RO* *+ depth* *+ RO × depth)	5,786.03	0.00	0.46	55	5,673.87
*R* ^*m*^(RO* *+ depth)	5,786.97	0.94	0.29	52	5,681.04
*R* ^*m*^(ROave* *+ Depthave* *+ *t*)	5,788.8	2.76	0.12	76	5,632.66
*R* ^*m*^(ROave* *+ *t*)	5,789.18	3.15	0.09	73	5,639.37
*R* ^*m*^(RO)	5,790.85	4.81	0.04	49	5,691.13

*Notes*

Models include the best supported structure for wetland dynamics, ψ^*m*^(Precipave* *+ *t*), and detection probability, δ(Vegave* *+ *t*). States (*m*) were modelled independently and covariates are abbreviated as follows: veg represents site‐specific vegetation cover that varies annually, depth is the maximum wetland depth, evap is total April to June evapotranspiration in mm, and RO is annual runoff in millimeters. The terms Depthave, Precipave, ROave, and Vegave represent the average value of these respective variables at sites over years and thus represent spatial variation only. Model selection information including Akaike's Information Criterion corrected for small sample size (AIC_c_), the difference in AIC_c_ from the top ranked model (ΔAIC_c_), model weights, number of parameters (*K*), and deviance are shown for each model.

**Figure 4:**
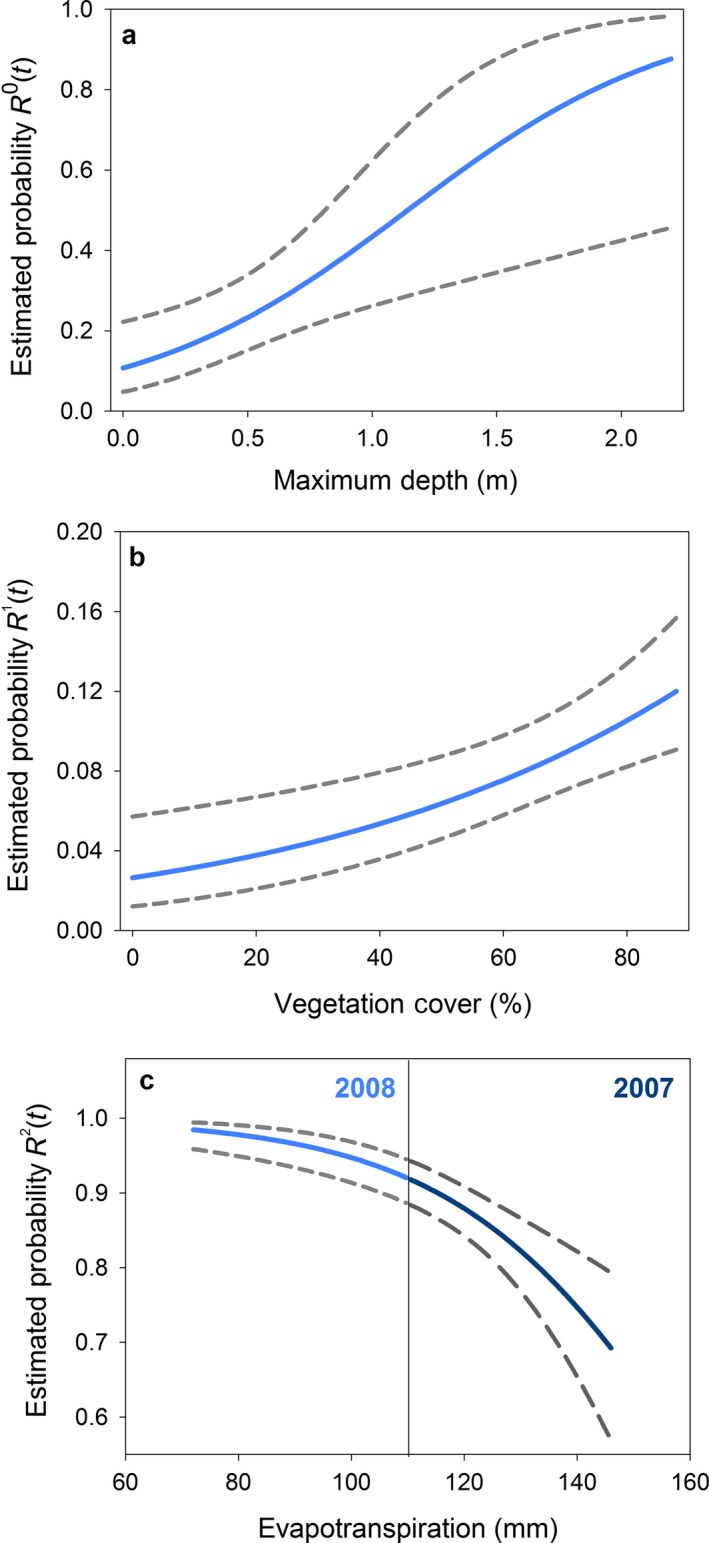
Relationships between conditional chorus frog breeding probabilities and climate drivers. Specifically, (a) the influence of maximum depth on conditional breeding colonization of dry wetlands in the prior year, R^0^(*t*); (b) the influence of vegetation cover on conditional breeding colonization of wet sites in the prior year, R^1^(*t*); and (c) the relationship between evapotranspiration and the probability of chorus frog breeding persistence at previously occupied wetlands, *R* ^2^(*t*). The range of evapotranspiration varied among years as evidenced by the nonoverlapping range between 2007 and 2008. Estimates are obtained from the best supported model, ψ^*m*^(Precipave* *+ *t*) R^*m*^(evap* *+ depth* *+ veg) δ(Vegave* *+ *t*), with covariates not included held at their decade‐long averages. The dashed lines represent approximate 95% confidence intervals.

Chorus frog breeding probabilities from the top model were related to the habitat characteristics of maximum depth and vegetation cover and to evapotranspiration (Table 3a). Conditional breeding probabilities of previously dry sites (*R* ^0^(*t*)) were higher in deeper wetlands with greater vegetation cover (Fig. 4a). Previously wet sites without breeding had higher colonization probabilities at sites with more vegetation cover (*R* ^1^(*t*); Fig. 4b), but little to no association with wetland maximum depth was identified. Conditional breeding persistence (*R* ^2^(*t*)) was positively related to vegetation cover and negatively associated with evapotranspiration, with a greater change demonstrated over the range of evapotranspiration values observed in dry years, such as 2007 (*R* ^2^(*t*); Fig. 4c). Unconditional breeding occupancy for chorus frogs ranged between 28% in 2007 to 44% in 2011 (Appendix  S1: Fig. S1). These estimates have good precision as evidenced by coefficients of variation ranging between 5% and 9%. Note that, in the dry years of 2007 and 2015, estimated breeding occupancy was at its lowest.

### Spotted frog dynamics

**Figure 5:**
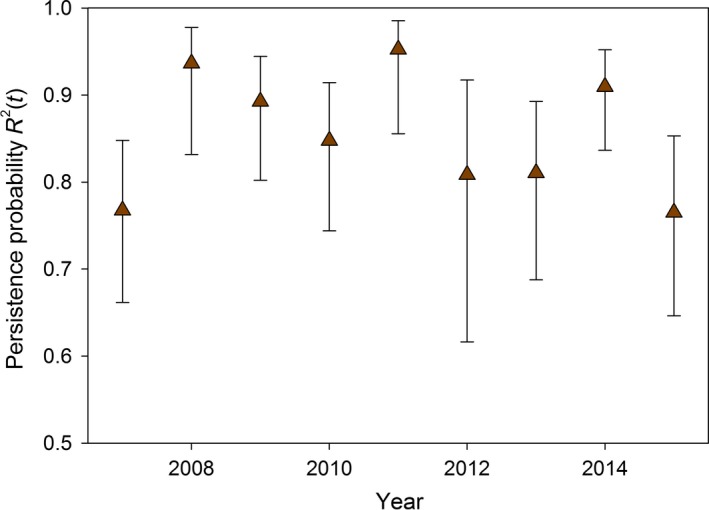
Model‐averaged breeding persistence probability, *R* ^2^(*t*), for spotted frogs at previously occupied wetlands. Error bars indicate approximate 95% confidence intervals.

Our top‐ranked model only garnered 46% of the model weight, so model‐averaged estimates are provided unless otherwise specified. Model‐averaged probability estimates of conditional breeding for previously dry sites, *R* ^0^(*t*), varied between 2% and 6% in any given year. Conditional breeding/colonization probabilities were similar for previously wet sites with no breeding (*R* ^1^(*t*) probabilities ranging between 3% and 7%). Wetlands with prior breeding were highly likely to support breeding in the following year with conditional breeding persistence probabilities, *R* ^2^(*t*), ranging 77% to 95% with the lowest estimates in dry years in 2007 and 2015 (Fig. 5).

**Figure 6:**
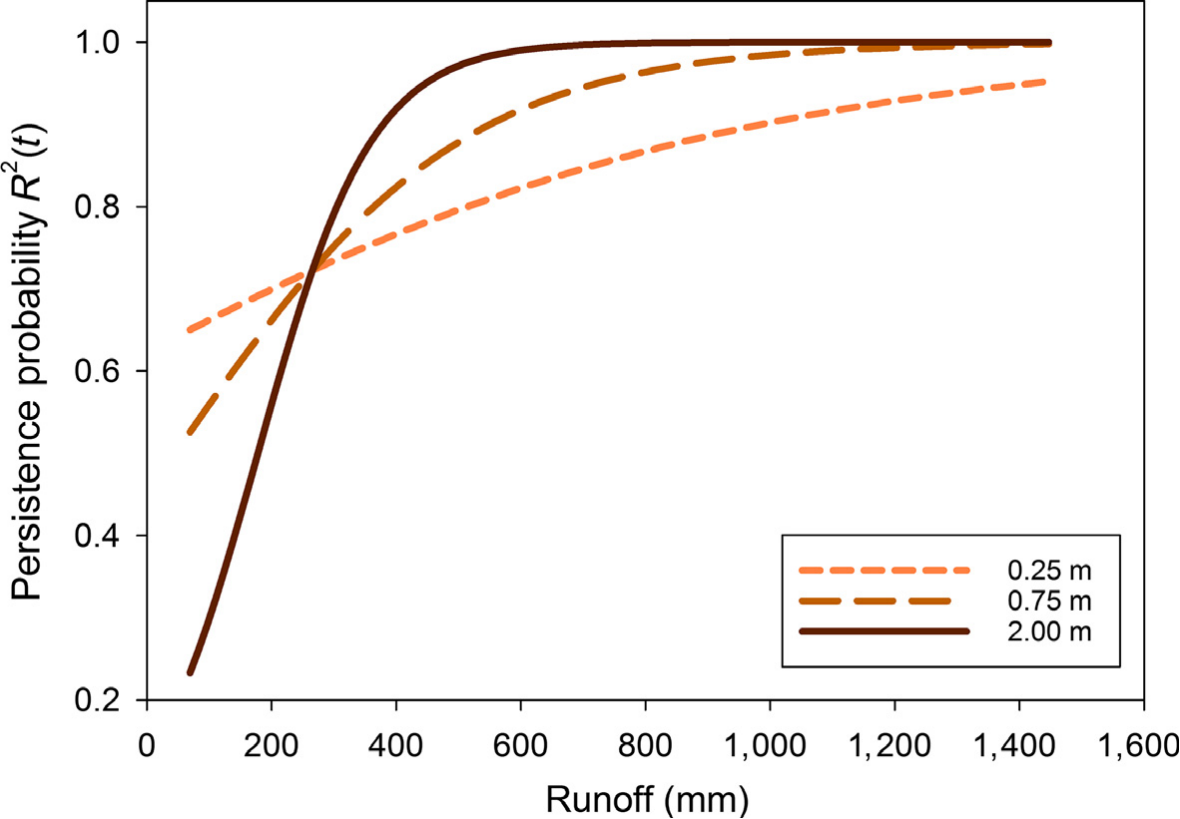
Estimated spotted frog breeding persistence probability, *R* ^2^(*t*), as related to runoff (RO) for three different maximum depths. Estimates are obtained from the best supported model, ψ^*m*^(Precipave* *+ *t*)*R* ^*m*^(RO **+ depth* *+ RO × depth)*p*(Vegave* *+ *t*).

The top two models indicated that spotted frog conditional breeding probabilities were related to runoff and maximum depth with or without an interaction (Table 3b). Positive associations with maximum depth and runoff were strongest for breeding persistence, *R* ^2^(*t*) (Fig. 6). Similarly, the interaction between maximum depth and runoff was most apparent in describing breeding persistence. As runoff increased, the probability of breeding persistence, *R* ^2^(*t*), increased regardless of maximum depth (Fig. 6). However, deeper wetlands had lower probabilities of breeding persistence than shallower wetlands with low runoff (<260 mm); with higher runoff (>260 mm), probabilities of breeding persistence were higher for deeper wetlands. Unconditional breeding occupancy for spotted frogs ranged 17% to 22% over the decade (Appendix  S1: Fig. S2). Note that the lowest estimates occur in the dry years of 2007 and 2015.

## Discussion

We use a multistate occupancy approach to jointly explore the effects of annual climate drivers on wetland and amphibian breeding dynamics over a 10‐yr period. Accounting for imperfect detection probability was important due to the interannual variability, due to different field crews and/or survey conditions over the decade. Estimated detection was notably low (49–60%) in 2010, but exceeded 70% in all other years. Not accounting for imperfect detection would have incorrectly supported a conclusion of a precipitous decline for both species in 2010. Hence, these results emphasize the importance of multiple surveys and accounting for imperfect detection in long term monitoring programs as has been emphasized by others (MacKenzie 2005, Schmidt 2005, Kery and Schmidt 2008).

Wetlands are dynamics habitats that respond to seasonal, annual, decadal, and multi‐decadal patterns in climatological drivers (McMenamin et al. 2008, Schook and Cooper 2014, Halabisky et al. 2016). Quantifying the variability in wetland drying is therefore fundamental to understanding dynamics in the occurrence of wetland dependent species (Zipkin et al. 2012) and in developing climate adaptation plans (Ryan et al. 2014). Similar to Ray et al. (2015), we found a 10‐fold range in the proportion of dry wetlands (2.4–29.2%).

The disparity in wetland inundation among years confirms the strong influence of climatological drivers on wetlands in our region. The three years with the highest runoff and precipitation values (2008, 2009, and 2011) had wetland inundation probabilities of over 85%. In contrast, the driest years had inundation probabilities between 5% and 18%. These patterns were consistent with covariate associations in lower ranked models that consisted of time‐varying climate covariates. Interestingly, we found a negative association between average local precipitation and the probability a wetland is suitable (wet) during the breeding season. We would expect wetlands that receive below average precipitation or runoff would typically be dry. In high moisture years, these sites are poised to hold water given sufficient input. In contrast, wetlands that are dry despite usually receiving above average precipitation must have other unmodeled factors (sensu Lee et al. 2015) that prevent them from retaining water in high moisture years. Our 10‐yr survey period did not include a prolonged drought, such as the region experienced in the early 2000s (see McMenamin et al. 2008, Sepulveda et al. 2015). In our time series, dry years were often followed by notably wet years (e.g., 2007–2008); therefore, we were unable to quantify the effects that multi‐year drought had on wetland dynamics. Ultimately, our expectation is that prolonged droughts forecasted by climate projections (Ryan et al. 2014) would likely result in few wetlands that transition from dry to wet during the drought period. Evaluating approaches to better model wetland dynamics will be necessary to prepare for future conditions under various climate change scenarios (Lee et al. 2015).

In addition to changing the habitat, prolonged drought would also likely decrease breeding occupancy for both anuran species, which over long periods, could result in local extinction especially for short‐lived species (Richter et al. 2003, Taylor et al. 2006). In other multi‐year studies, wetland drying during drought years resulted in decreased occupancy of pond‐breeding amphibians (Walls et al. 2013, Mac Nally et al. 2017). Climate change is predicted to increase the hydrologic variability of wetlands (Grant et al. 2013) and transform a greater percentage of wetlands from permanent and semi‐permanent status to an ephemeral status (Lee et al. 2015). These predicted changes may lower occupancy rates synchronously across obligate aquatic species, but species in unpredictable environments with faster life histories such as chorus frogs, may exhibit adaptive reproduction and dispersal strategies as compared to species from less variable environments (Cayuela et al. 2016a, 2016b).

Breeding dynamics for chorus frogs indicate that ephemeral wetlands (i.e., those that may be dry in years with low runoff and precipitation) are often colonized when inundated (Fig. 3). This result is consistent with other studies that have demonstrated higher dispersal rates for shorter‐lived species in temporally variable environments (Stevens et al. 2013). Previously dry sites with greater maximum depth and vegetation cover were more likely to be colonized than shallower sites with less vegetation, but vegetation cover appeared to be more important for breeding at wetlands that retained water. Vegetation provides numerous benefits for larval amphibians. First, chorus frogs and other anurans use vegetative braces for depositing egg masses (Scherer et al. 2012). Free‐floating and submersed vegetation can also significantly increase surface water temperatures relative to unvegetated ponds (Dale and Gillespie 1977) and amphibian larvae show a preference for warmer waters where development rates may be faster (Smith‐Gill and Berven 1979). Vegetation also provides larvae refugia from predators (Formanowicz and Bobka 1989) and may serve as foraging locations for larvae that consume epiphytes (Woodward and Mitchell 1992).

We found a pronounced decline in chorus frog breeding persistence probabilities when evapotranspiration was high. During hot and dry years, summer evapotranspiration exceeds precipitation and contributes to a shortening of wetland hydroperiods, depletion of available water on the landscape, and a significant loss of available wetland habitat (Schook and Cooper 2014). Chorus frogs are particularly vulnerable to evapotranspiration‐induced wetland drying due to breeding habitat preference for wet meadows, ephemeral wetlands, and the shallower portions of permanent wetlands (Koch and Peterson 1995). Although evapotranspiration rates may not be enough to cause the complete drying of deeper ponds in our study area, elevated evapotranspiration rates in dry years may exclude chorus frogs from important reproductive locations even within permanent ponds (Amburgey et al. 2016).

Spotted frogs had lower and less variable estimates of breeding occupancy in comparison to chorus frogs, relying mostly on permanent sites with prior breeding. The interaction between maximum depth and runoff for spotted frogs indicates that breeding persistence in shallow wetlands was lower across most runoff levels than intermediate and deep wetlands. Deeper wetlands had almost 100% breeding persistence across a range of runoff levels (Fig. 6). The hydroperiods of larger and deeper wetlands are generally less sensitive to annual variations in meteorological conditions and are able to withstand extended periods of drought better than shallower wetlands (Ryan et al. 2014, Lee et al. 2015). Within Yellowstone National Park, deeper groundwater‐ or surface‐water‐connected wetlands with relatively stable water levels have persisted despite widespread drying of wetlands across periods of drought (Schook and Cooper 2014). The strong relationship between wetland maximum depth, runoff, and spotted frog breeding persistence is consistent with habitat characteristics that contribute to population persistence and growth across the species range (Hossack et al. 2013). High breeding persistence probabilities of spotted frogs (and chorus frogs) are consistent with our prediction and echo the importance of maintaining permanent wetlands, especially when one considers that historical breeding is a strong indicator of future breeding for both anuran species.

Climate projections indicate a future with higher temperatures, earlier snowmelt and shorter hydroperiods (Ryan et al. 2014). McCaffery and Maxell (2010) suggested that ectotherms living at high elevations may benefit from global warming as long as the changes do not negatively affect habitat (e.g., reduced habitat heterogeneity). In their montane wetlands, spotted frogs had higher breeding probabilities following less severe winters (breeding probability was measured as the proportion of egg masses to adult females). Although our definitions of breeding probability differ, we found that wetland habitat was reduced in years with less moisture (i.e., wetland drying in 2007 and 2015), which in turn reduced breeding occupancy. The absence of breeding may be from adult females opting not to breed in poor wetland conditions, or they may disperse to other wetlands that have suitable habitat as described by Cayuela et al. (2016b). In either case, our expectation that chorus frogs would have higher colonization probabilities in ephemeral wetlands was supported in accordance with their position along a fast‐slow continuum (sensu Sæther 1987).

The strengths of the current work are multifold. Our use of multistate models have provided insight on the differential use of ephemeral wetlands for breeding by two anuran species and allowed us to quantify state transitions based on climate drivers and habitat characteristics. We have employed consistent survey methods that account for imperfect breeding detection for a decade across a large geographic area mostly removed from anthropogenic influences (McMenamin et al. 2008). With an average of 291 wetlands surveyed each year, our sample size is notably larger than most studies (Green et al. 2013, Fellers et al. 2015, Groff et al. 2017) and demonstrates the reward that long term monitoring of natural resources can offer when using an a priori multiple hypothesis approach to describe system dynamics. Further, the current multistate approach has the advantage of estimating the percentage of dry wetlands each year and allows for an expansion of additional wetland states. For example, in the event of prolonged drought, one could have several dry states defined by the number of consecutive dry years prior to holding water, thereby allowing one to investigate the cumulative effects of drought on conditional breeding probabilities. Ultimately, our conclusions echo those of McCaffery et al. (2014) and Ryan et al. (2014) in which they clarify that habitat heterogeneity (the presence of both ephemeral, semi‐permanent, and permanent wetlands) or a portfolio of wetland types is likely important in sustaining breeding amphibian populations. The modeling approach described here provides necessary insight on the current and previous year drivers of amphibian occurrence in the Greater Yellowstone Area. This approach may prove equally valuable in evaluating determinants of species occurrence for other species that depend on wetlands or other dynamic habitats.

## Data Availability

Data are available from the Integrated Resource Management Applications (IRMA) Data Store at https://irma.nps.gov/DataStore/Reference/Profile/2257008.
