# Supplementary material for: Multistate occupancy modeling improves understanding of amphibian breeding dynamics in the Greater Yellowstone Area
Source: Ecol Appl. 2019 Jan 2;29(1):e01825. doi: 10.1002/eap.1825 (PMC7017861; doi:10.1002/eap.1825)

William R. Gould, Andrew M. Ray, Larissa L. Bailey, David Thoma, Rob Daley and Kristin Legg. Multistate occupancy modeling improves understanding of amphibian breeding dynamics in the Greater Yellowstone Area. Ecological Applications

Table S1. Multi-season multistate analysis (2006-2015) for boreal chorus frogs at wetland sites within the Greater Yellowstone Ecosystem. Wetland and breeding states (*m*) are modelled independently, i.e., as an interaction with the covariate and/or year combination. The term '*t*' represents year-specific effects. Covariates are abbreviated as follows: 'veg' represents percent vegetation cover, 'depth' is the maximum wetland depth, 'precip' is precipitation (April to June) in mm, 'evap' is evapotranspiration (April to June) in mm, and 'RO' is yearly runoff in mm. The terms 'Depthave', 'Precipave' 'ROave', and 'Vegave' represent the average value of these respective variables at sites over years and thus represent spatial variation only. The term 'Lag' represents a one-year lag in the variable.

Model	AICc	ΔAICc	AICc Weight	K	Deviance
{ψ^{m}(Precipave+t)R^{m}(evap+depth+veg)δ(Vegave+t)}	6784.41	0.00	0.94	55	6672.25
{ψ^{m}(Precipave+t)R^{m}(evap+veg)δ(Vegave+t)}	6789.89	5.48	0.06	52	6683.96
{ψ^{m}(Precipave+t)R^{m}(Evapave+Depthave+Vegave+t)δ(Vegave+t)}	6800.77	16.36	0.00	79	6638.30
{ψ^{m}(Precipave+t)R^{m}(depth+veg)δ(Vegave+t)}	6808.14	23.74	0.00	52	6702.21
{ψ^{m}(Precipave+t)R^{m}(evap+depth)δ(Vegave+t)}	6809.94	25.54	0.00	52	6704.01
{ψ^{m}(Precipave+t)R^{m}(evap)δ(Vegave+t)}	6810.17	25.77	0.00	49	6710.46
{ψ^{m}(Precipave+t)R^{m}(Evapave+Vegave+t)δ(Vegave+t)}	6811.69	27.29	0.00	76	6655.56
{ψ^{m}(Precipave+t)R^{m}(veg)δ(Vegave+t)}	6815.79	31.39	0.00	49	6716.08
{ψ^{m}(Precipave+t)R$^{0=1}$(t)δ(Vegave+t)}	6821.63	37.22	0.00	61	6696.97
{ψ^{m}(Precipave+t)R^{m}(Depthave+Vegave+t)δ(Vegave+t)}	6823.96	39.55	0.00	76	6667.82
{ψ^{m}(Precipave+t)R^{m}(Vegave+t)δ(Vegave+t)}	6824.55	40.14	0.00	73	6674.73
{ψ^{m}(Precipave+t)R^{m}(t)δ(Vegave+t)}	6827.02	42.62	0.00	70	6683.52
{ψ^{m}(Evapave+t)R^{m}(t)δ(Vegave+t)}	6828.72	44.32	0.00	70	6685.22
{ψ^{m}(t)R^{m}(t)δ(Vegave+t)}	6829.88	45.47	0.00	67	6692.67
{ ψ^{m}(Precipave+t)R^{m}(Evapave+Depthave+t)δ(Vegave+t)}	6830.46	46.06	0.00	76	6674.33
{ ψ^{m}(Precipave+t)R^{m}(.)δ(Vegave+t)}	6830.72	46.31	0.00	46	6737.21
{ ψ^{m}(Precipave+t)R^{m}(depth)δ(Vegave+t)}	6830.75	46.35	0.00	49	6731.04
{ ψ^{m}(ROave+t)R^{m}(t)δ(Vegave+t)}	6831.16	46.76	0.00	70	6687.66
{ ψ^{m}(Precipave+Evapave+t)R^{m}(t)δ(Vegave+t)}	6840.59	56.19	0.00	73	6690.78
{ ψ^{m}(ROave+Evapave+t)R^{m}(t)δ(Vegave+t)}	6842.44	58.03	0.00	73	6692.63
{ψ^{m}(t)R^{m}(t)δ(t)}	6846.96	62.55	0.00	66	6711.84
{ψ$^{1=2}$(t)R^{m}(t)δ(Vegave+t)}	6847.14	62.73	0.00	58	6728.74
{ψ^{m}(t)R^{m}(t)δ(veg)}	6849.02	64.61	0.00	58	6730.62
{ψ^{m}(Precipave+t)R^{m}(Evapave+t)δ(Vegave+t)}	6849.55	65.14	0.00	73	6699.74
{ψ^{m}(Precipave+t)R$^{0=1}$(.)δ(Vegave+t)}	6871.57	87.17	0.00	45	6780.13
{ψ^{m}(t)R^{m}(t)δ(.)}	6884.32	99.91	0.00	57	6768.00
{ψ^{m}(Precipave+t)R^{m}(Depthave+t)δ(Vegave+t)}	6893.71	109.31	0.00	73	6743.90
{ψ^{m}(precip+evap)R^{m}(t)δ(Vegave+t)}	7028.52	244.11	0.00	49	6928.80
{ψ^{m}(precip)R^{m}(t)δ(Vegave+t)}	7047.32	262.92	0.00	46	6953.81
{ψ^{m}(RO+evap)R^{m}(t)δ(Vegave+t)}	7056.66	272.26	0.00	49	6956.95

$\{\psi^{m}(evap)R^{m}(t)\delta(Vegave+t)\}$	7071.16	286.75	0.00	46	6977.65
$\{\psi^{m}(RO)R^{m}(t)\delta(Vegave+t)\}$	7094.09	309.69	0.00	46	7000.58
$\{\psi^{m}(precipLag)R^{m}(t)\delta(Vegave+t)\}$	7130.31	345.91	0.00	46	7036.80
$\{\psi^{m}(.)R^{m}(t)\delta(Vegave+t)\}$	7154.53	370.12	0.00	43	7067.21
$\{\psi^{m}(evapLag)R^{m}(t)\delta(Vegave+t)\}$	7155.50	371.09	0.00	46	7061.98
$\{\psi^{1=2}(.)R^{m}(t)\delta(Vegave+t)\}$	7174.63	390.22	0.00	42	7089.37
$\{\psi^{m}(.)R^{m}(t)\delta(Vegave+t)\}$	7184.01	399.60	0.00	43	7096.69
$\{\psi^{m}(Precipave+t)R^{1=2}(.)\delta(Vegave+t)\}$	7837.53	1053.1	0.00	45	7746.08
$\{\psi^{m}(Precipave+t)R^{1=2}(t)\delta(Vegave+t)\}$	7841.46	1057.0	0.00	61	7716.80

Table S2. Multi-season multi-scale model set (2006-2015) for spotted frogs at wetland sites within the Greater Yellowstone Ecosystem. Wetland and breeding states (*m*) are modelled independently, i.e., as an interaction with the covariate and/or year combination. The term 't' represents a year-specific effect. Covariates are abbreviated as follows: 'veg' represents vegetation cover, 'depth' is the maximum wetland depth in m, 'precip' is precipitation (April-June) in mm, 'evap' is evapotranspiration (April-June) in mm, and 'RO' is yearly runoff in mm. The terms 'Depthave', 'Precipave' 'ROave', and 'Vegave' represent the average value of these respective variables at sites over years and thus represent spatial variation only. The term 'Lag' represents a one-year lag in the variable.

Model	AICc	ΔAICc	AICc Weight	K	Deviance
{ψ^{m}(Precipave+t)R^{m}(RO+depth+RO*depth)p(Vegave+t)}	5786.03	0.00	0.46	55	5673.87
{ψ^{m}(Precipave+t)R^{m}(RO+depth)p(Vegave+t)}	5786.97	0.94	0.29	52	5681.04
{ψ^{m}(Precipave+t)R^{m}(ROave+Depthave+t)p(Vegave+t)}	5788.80	2.76	0.11	76	5632.66
{ψ^{m}(Precipave+t)R^{m}(ROave+t)p(Vegave+t)}	5789.18	3.15	0.09	73	5639.37
{ψ^{m}(Precipave+t)R^{m}(RO)p(Vegave+t)}	5790.85	4.81	0.04	49	5691.13
{ψ^{m}(Precipave+t)$R^{0=1}$(t)p(Vegave+t)}	5800.49	14.46	0.00	61	5675.83
{ψ^{m}(Precipave+t)R^{m}(depth)p(Vegave+t)}	5808.97	22.94	0.00	49	5709.26
{ψ^{m}(Precipave+t)$R^{0=1}$(.)p(Vegave+t)}	5810.34	24.31	0.00	45	5718.89
{ψ^{m}(Precipave+t)R^{m}(.)p(Vegave+t)}	5812.38	26.35	0.00	46	5718.87
{ψ^{m}(Precipave+t)R^{m}(t)p(Vegave+t)}	5813.73	27.69	0.00	70	5670.22
{ψ^{m}(t)R^{m}(t)p(Vegave+t)}	5814.29	28.26	0.00	67	5677.08
{ψ^{m}(t)R^{m}(t)p(t) }	5815.21	29.17	0.00	66	5680.09
{ψ^{m}(Precipave+t)R^{m}(Depthave+t)p(Vegave+t)}	5816.63	30.59	0.00	73	5666.81
{ψ^{m}(ROave+t)R^{m}(t)p(Vegave+t)}	5817.09	31.05	0.00	70	5673.58
{ψ^{m}(Precipave+Evapave+t)R^{m}(t)p(Vegave+t)}	5817.43	31.40	0.00	73	5667.62
{ψ^{m}(Evapave+t)R^{m}(t)p(Vegave+t)}	5818.72	32.68	0.00	70	5675.21
{ψ^{m}(ROave+Evapave+t)R^{m}(t)p(Vegave+t)}	5819.38	33.34	0.00	73	5669.57
{ψ^{m}(t)R^{m}(t)p(veg) }	5831.45	45.42	0.00	58	5713.05
{ψ^{m}(t)R^{m}(t)p(.)}	5835.67	49.63	0.00	57	5719.35
{$\psi^{1=2}$(t)R^{m}(t)p(Vegave+t)}	5861.39	75.36	0.00	58	5742.99
{ψ^{m}(precip+evap)R^{m}(t)p(Vegave+t)}	6005.32	219.29	0.00	49	5905.61
{ψ^{m}(precip)R^{m}(t)p(Vegave+t)}	6026.82	240.78	0.00	46	5933.31
{ψ^{m}(RO+evap)R^{m}(t)p(Vegave+t)}	6032.36	246.32	0.00	49	5932.64
{ψ^{m}(evap)R^{m}(t)p(Vegave+t)}	6047.01	260.98	0.00	46	5953.50
{ψ^{m}(RO)R^{m}(t)p(Vegave+t)}	6074.71	288.68	0.00	46	5981.20
{ψ^{m}(precipLag)R^{m}(t)p(Vegave+t)}	6118.87	332.83	0.00	46	6025.36
{ψ^{m}(evapLag)R^{m}(t)p(Vegave+t)}	6143.27	357.24	0.00	46	6049.76
{ψ^{m}(.)R^{m}(t)p(Vegave+t)}	6148.58	362.55	0.00	43	6061.26
{$\psi^{1=2}$(.)R^{m}(t)p(Vegave+t)}	6192.10	406.06	0.00	42	6106.84
{ψ^{m}(Precipave+t)$R^{1=2}$(t)p(Vegave+t)}	6559.69	773.65	0.00	61	6435.03
{ψ^{m}(Precipave+t)$R^{1=2}$(.)p(Vegave+t)}	6561.60	775.56	0.00	45	6470.15

Fig. S1. Unconditional probability that a wetland supports breeding chorus frogs (a derived parameter) over a 10-yr period within Yellowstone and Grand Teton national parks. Estimates obtained from the top ranked model, ψ^{m}(Precipave+*t*)R^{m}(evap+depth+veg)δ(Vegave+*t*), using mean values of all covariates for each year. Error bars indicate approximate 95% confidence intervals.

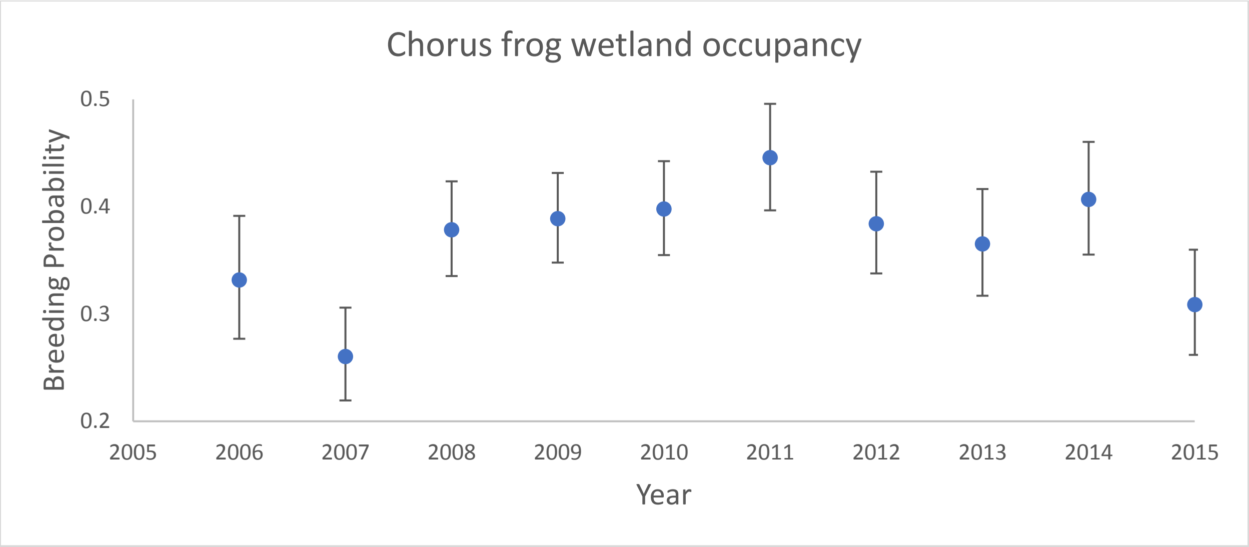

Fig. S2. Unconditional probability that a wetland supports breeding spotted frogs over a 10-yr period within Yellowstone and Grand Teton national parks. These model-average estimates are derived using the mean values of all covariates for each year. Error bars indicate approximate 95% confidence intervals.

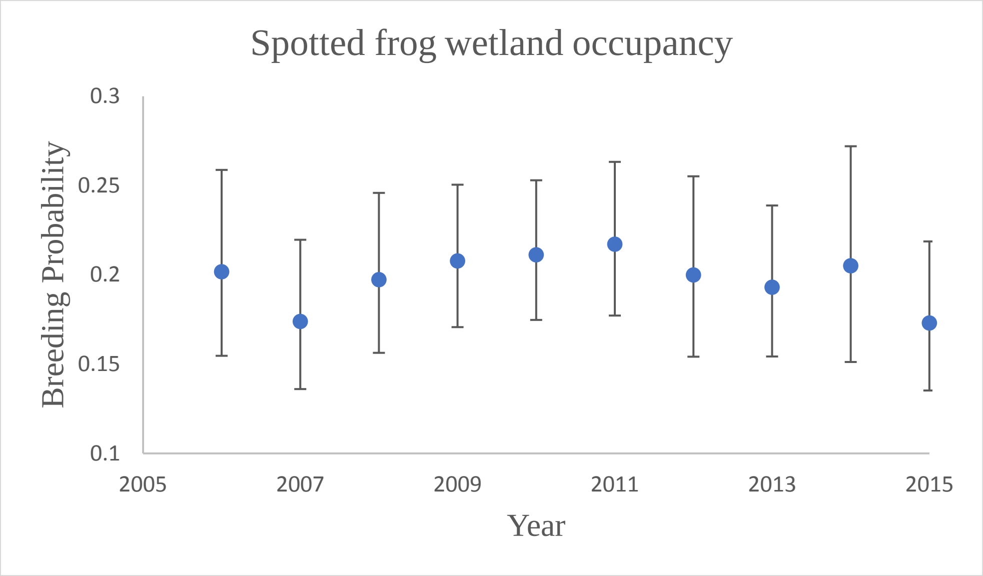

Supplement: Supplementary file 1 [file EAP-29-e01825-s001.pdf]
